# Isolated splenic metastases from rectal carcinoma 5 years after surgery: Case report

**DOI:** 10.1097/MD.0000000000032493

**Published:** 2023-01-13

**Authors:** Jie Xu, Yanan He, Yu Cai, Yi Huang, Yuanyang He

**Affiliations:** a Hepatobiliary Surgery, Jiangyou People’s Hospital, Sichuan Province, China; b North Sichuan Medical College, Nanchong, Sichuan Province, China; c Hepatobiliary Surgery, The Third Hospital of Mianyang·Sichuan Mental Health Center, Sichuan Province, China.

**Keywords:** metastatic tumor, rectal cancer, spleen

## Abstract

Splenic malignancies are mostly lymphocytic tumors and splenic metastases are rarer.^[1]^ According to reports, the most common source of splenic metastases include melanoma, tumors of the breast, lung, ovary, colon, stomach, and pancreas.^[2,3]^

**Patient concerns:**

This paper reports a 41-year-old male patient who underwent a successful resection of low rectal cancer in our hospital 5 years ago.

**Diagnosis:**

Three months ago, computed tomography scan revealed a tumor in the spleen, considered as an isolated metastasis.

**Interventions:**

The patient underwent splenectomy and postoperative pathological examination confirmed metastatic adenocarcinoma.

**Outcomes:**

The patient was followed up for 3 months after surgery, there was no abdominal metastasis or recurrence.

**Conclusion:**

The splenic metastasis from rectal carcinoma 5 years after surgery is rare. If it is a solitary splenic metastasis, splenectomy can effectively improve the prognosis of patients. We review the literature and report this case.

## 1. Introduction

Splenic malignancies are mostly lymphocytic tumors and splenic metastases are rarer.^[1]^ According to reports, the most common source of splenic metastases include melanoma, tumors of the breast, lung, ovary, colon, stomach, and pancreas.^[2,3]^

## 2. Case report

Patient, male, 41 years old. The patient was admitted to our hospital in May 2016 due to repeated abdominal pain and discomfort, changes in stool shape, and bleeding. Colonoscopy suggests: rectal eminence venereal changes with stenosis. Computed tomography (CT): there was a high possibility of neoplastic lesions in sigmoid colon and upper rectum; irregular thickening of the middle and lower rectum wall; multiple enlarged lymph nodes are seen in the abdominal cavity, pelvic cavity, and bilateral inguinal area. Pathological diagnosis: adenocarcinoma. After 3 cycles of fluorouracil + calcium leucorin + oxaliplatin chemotherapy, the patient underwent low rectal anterior resection and knot-rectal anastomosis in August 2016. Postoperative pathological: rectal ulcerated moderately differentiated adenocarcinoma invaded serous layer, metastasis was observed in 11/14 mesenteric lymph nodes, tumor stage (T4N2M0). Four cycles of fluorouracil + calcium leucorin + oxaliplatin chemotherapy were performed postoperatively, followed by concurrent radiotherapy.

**Figure 1.**
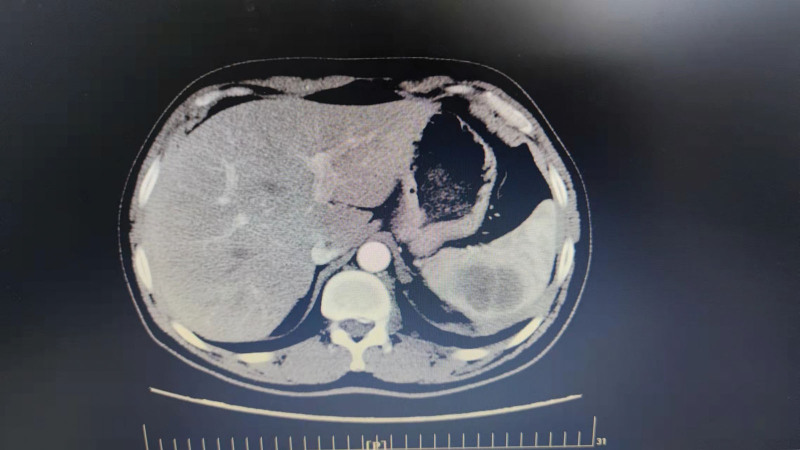
Computed tomography (CT) scan of a patient with splenic metastasis from rectal carcinoma.

Reexamination was conducted every 3 months and no abnormality was found. Abdominal CT of the patient on March 15, 2021 indicated a low-density mass shadow in the spleen, about 3.1 cm × 4.2 cm in size. Abdominal CT: splenic mass, metastatic tumor was considered; the adjacent left diaphragmatic surface; swollen lymph nodes adjacent to the abdominal aorta. So the patient underwent 10 cycles of irinotecan + fluorouracil + leucovorin chemotherapy and bevacizumab treatment. CT (Fig. 1) was reexamined after chemotherapy: spleen mass was considered metastasis; swollen abdominal lymph node was not observed. The patient underwent laparoscopic splenectomy on September 8, 2011. During the operation, it was found that the spleen capsule and the diaphragm were densely adhered, and part of the diaphragm was removed. Postoperative pathology: adenocarcinoma; check out the signet ring cells; there are no tumor cells in the diaphragm. After 3 months of follow-up, the abdominal CT showed no swollen intraperitoneal lymph node and new lesions.

## 3. Discussion

Splenic tumors include primary and metastatic tumors. Primary tumors include hemangioma, lymphoma, lymphangioma, hamartoma, coastal hemangioma, hemangioendothelioma, hemangiepidooma, and hemangiosarcoma.^[4]^ Primary cystadenocarcinoma of the spleen is very rare.^[5]^ Autopsy reports from a large number of cancer patients showed splenic metastasis rates ranging from 2.3 to 7.1%.^[6,7]^ Splenic metastases is usually a late-stage performance of cancer patients.^[8]^ It is rare for non-hematological diseases to metastasize to the spleen, and the primary tumors are mostly melanoma, breast, lung, ovary, colon, stomach, and pancreas tumors.^[2,3]^ A study found that 20.9% of patients in 29,364 patients with carcinoma had metastases, but only 59 patients had splenic metastases (accounting for 0.002% of 29,364 patients).^[9]^

Colorectal cancer is the third most common cancer in the world, and about 20% of patients present with metastatic disease.^[10,11]^ At present, distant recurrence is the main cause of cancer-related death in patients with rectal cancer.^[12]^ The liver is the most common site of distant metastasis, other common sites include local lymph nodes, lungs, and peritoneum, and less common sites include the brain and bone.^[13]^ The spleen is a rare site for colorectal cancer metastasis. An autopsy found that 21 (2%) of 1019 patients with colorectal cancer were found to have splenic metastases, and none of them were isolated splenic metastases.^[7]^ A recent paper found 34 cases of isolated splenic metastasis after colorectal cancer surgery in PubMed database, including 28 cases of heterochronous metastasis.^[14]^

The spleen is a highly vascularized organ with a large number of reticuloendothelial cells, but metastatic lesions are rare.^[15]^ The reason for the low incidence of splenic metastases is unclear, and several hypotheses have been proposed from anatomic, physiological, and immunological perspectives: there is an acute angle at the beginning of the splenic artery that restricts tumor cell metastasis to the spleen; the spleen prevents tumor cells implanting into vascular endothelial cells through rhythmic contraction; lymphocytes and macrophages can prevent tumor cell implantation and proliferation in the spleen, or can inhibit tumor cell survival; and the parenchyma of the spleen lacks the input of lymphatic vessels, and only a few rare lymphatic vessels are confined to the splenic capsule.^[16–18]^

This article introduces a case of heterogeneous and isolated splenic metastasis. I believe that this tumor is formed by lymphatic metastasis. The reasons are as follows: the spleen is located in the upper left abdomen and is far away from the rectum, so do not consider for the time being; due to the special anatomical structure of the artery, it is not considered; if tumor cells retrograde into the splenic vein via the inferior mesenteric vein and colonize the spleen, liver metastasis should occur first, and then systemic metastasis should occur. No liver metastasis was found in this patient 5 years after operation, so we believe that it is not through the splenic vein; and there is lymphatic input under the splenic capsule, and the tumor in this case is located just under the capsule. The patient found enlarged celiac lymph nodes 6 months before splenectomy, but no further examination due to the lack of positron emission CT examination. After systemic chemotherapy and targeted therapy, no enlarged lymph nodes were found, but the splenic tumor was enlarged. To sum up, we believe that this patient developed splenic metastases through lymphatic metastasis.

Because of the small number of cases, there is no randomized trial to verify the therapeutic effect of splenectomy on isolated splenic metastasis of colorectal cancer.

At present, most of the cases in the literature used splenectomy to treat isolated splenic metastases, which seems to be an effective method for the treatment of splenic metastases.^[9]^ If left untreated, splenic metastasis may lead to splenic rupture and forced surgical treatment. The survival after splenectomy is not clear, and the data reported in the literature show that they may survive for up to 7 years.^[19]^

## 4. Conclusion

Splenic metastasis derived from rectal cancer is rare, and most patients are found in regular reexamination of imaging. If it is a solitary splenic metastasis, splenectomy can effectively improve the prognosis of patients.

## Author contributions

**Data curation:** Jie Xu.

**Formal analysis:** Yanan He, Yu Cai.

**Investigation:** Yanan He, Yi Huang.

**Methodology:** Jie Xu, Yu Cai, Yi Huang.

**Visualization:** Yanan He, Yu Cai.

**Writing – original draft:** Jie Xu, Yuanyang He.

**Writing – review & editing:** Yanan He, Yi Huang, Yuanyang He.
